# Normality in Analytical Psychology

**DOI:** 10.3390/bs3040647

**Published:** 2013-11-21

**Authors:** Steve Myers

**Affiliations:** Centre for Psychoanalytic Studies, University of Essex, Wivenhoe Park, Colchester CO4 3SQ, UK; E-Mail: spmyers@teamtechnology.co.uk

**Keywords:** Jung, analytical psychology, normality, individuation, collectivity, Foucault, Freud

## Abstract

Although C.G. Jung’s interest in normality wavered throughout his career, it was one of the areas he identified in later life as worthy of further research. He began his career using a definition of normality which would have been the target of Foucault’s criticism, had Foucault chosen to review Jung’s work. However, Jung then evolved his thinking to a standpoint that was more aligned to Foucault’s own. Thereafter, the post Jungian concept of normality has remained relatively undeveloped by comparison with psychoanalysis and mainstream psychology. Jung’s disjecta membra on the subject suggest that, in contemporary analytical psychology, too much focus is placed on the process of individuation to the neglect of applications that consider collective processes. Also, there is potential for useful research and development into the nature of conflict between individuals and societies, and how normal people typically develop in relation to the spectrum between individuation and collectivity.

## 1. Introduction

C.G. Jung’s interest in the subject of normality waxed and waned during the second half of his life, but at no time did he assemble a coherent account of his multifaceted view. In 1926, he introduced the word *normal* into the title of the first of his Two Essays [1], and in the 1930s he cited the lack of inclusion of normal psychology in Freud’s thinking as one of the main factors in their separation [2]. He also used the term *complex psychology* to make his theory more relevant to general psychology [3]. However, Jung had some esoteric values—seeing normal people as of lesser value ([1], p.149) and having a poor view of groups and society ([1], pp. 152–154)—and in 1943 he took the word *normal* back out of the Two Essays title ([1], pp. 7–8). However, when Jung sketched out his wish-list for the future of analytical psychology in 1948, normal psychology was one of the main topics he highlighted:

In normal psychology, the most important subjects for research would be the psychic structure of the family in relation to heredity, the compensatory character of marriage and of emotional relationships in general. A particularly pressing problem is the behaviour of the individual in the mass and the unconscious compensation to which this gives rise.

[4]

Although Jung continues by suggesting a broad range of further applications—including the humanities, religion, science, and other areas of psychology—this paper is going to focus on the “pressing problem” that he highlighted. Both aspects of the problem—the individual-mass relationship and unconscious compensation—feature heavily in many of Jung’s writings about normality. Also, this problem is close to some of the main themes in analytical psychology, such as individuation, rapprochement between consciousness and the unconscious, the union of opposites, the transcendent function, *etc.*

For Jung, normality is finding one’s needs being met in the situations of daily life ([1], p. 55)—a deceptively simple description that belies a sophisticated understanding. In order to explore what this entails, and its implications, we first have to differentiate Jung’s understanding from the related disciplines from which it emerged, *i.e.*, psychoanalysis and mainstream psychiatry/psychology. Also, we need to examine Foucault’s criticisms of normality to establish whether they might undermine Jung’s concepts and their application.

## 2. Foucault

### 2.1. Foucault and Psychoanalysis

Foucault did not make any comment about Jung’s theories, but provided a critique of the use of normality in mainstream psychiatry, psychology and psychoanalysis. Mainstream definitions of normality, where they are accepted, are usually derived from the mass. For example, one psychology textbook describes “the idea of normality [as] socially constructed and a contested notion” [5]. Norms naturally develop in group situations, which individuals then use to govern their own personal behaviour [6]. These norms are more powerful in shaping individual behaviour than education [7]. Foucault’s criticism was targeted primarily at this type of norm, *i.e.*, “order defined by natural and observable processes” [8]. He argued that those in authority, such as psychiatrists, used norms alongside artificial regulation to impose treatment, exclusion or imprisonment on those who were decreed as being abnormal. For Foucault, treatments were not based on medical science because “Madness-Disorder relations centred on the theme of social and moral order” [9]. He saw the advent of psychoanalysis as bringing a change in the style rather than the substance of treatment:

What we call psychiatric practice is a certain moral tactic contemporary with the end of the eighteenth century...all nineteenth century psychiatry really converges on Freud [who] demystified the asylum...[but] to the doctor, Freud transferred all the structure Pinel and Tuke had set up in confinement.

([9], pp. 164–165)

Foucault’s criticism was that “the formulations [Freud] hears are always those of transgression” ([9], p. 152) (of the limits of standard knowledge and experience [10]). He saw the psychoanalytic doctor-patient interaction as a continuation of normalisation processes—using the tactics of observation, silence, unspoken judgement and the mirroring of madness. However, Foucault’s argument has itself been criticised: he “could never give up the temptation to valorize transgression [so] was unable to pursue the dialogue with unreason in a systematic way” [11]. In his archaeology of the ideas of madness and their discursive formation, Foucault looked only at the context in which psychoanalysis had developed and he didn’t engage with Freud’s actual theory [12]. 

The relevance of Foucault’s criticisms therefore start to unfold with the advent of psychoanalysis because it was Foucault, not Freud, who could only hear formulations based on transgression. This meant he only saw Freud’s understanding of normality as a social construct and he failed to recognise that Freud’s understanding of pathology was based on intra-psychic processes rather than the transgression of social norms. Freud viewed a normal person as someone for whom the preconscious and the unconscious were not in conflict [13] or who was “free from neurosis” [14]. These attributes were not (as Foucault’s criticism implies) prevalent within the population and which some transgressed, but rather they were attributes that *no one* possessed. Also, even though Freud wrote about normality and claimed psychoanalysis “developed into a psychology of normal mental life” [15], his starting point was pathology (e.g., see [16]) which meant his understanding went in the opposite direction to Foucault’s claims. Whereas Foucault saw Freud as using social norms to define what was pathological, Freud in fact used, what could be viewed as, abnormal phenomena in order to understand normal ones (e.g., see [17]).

However, there have been some post-Freudian developments that could bring some psychoanalytic conceptions of normality back into the scope of Foucault’s criticisms. Abraham, Jones, Glover, Gitelson, Klein, Krapf, Anna Freud and others have all developed their own thinking on the subject, taking the concept of psychoanalytic normality in several directions. Kubie described the main strands as being phenomenological, sociological, and ontogenetic but, to provide a better differentiation of psychological health from illness, he added his own criteria of flexibility and (similar to Freud) intra-psychic harmony [18]. Offer and Sabshin suggested there were four main psychoanalytic understandings of normality [19]: an average, such as Glover’s “social standards of adaptation” [20]; a disease, such as Gitelson’s view that it is living behind a façade of adaptation [21]; a process, such as Anna Freud’s stages of child development; and a healthy (but unobtainable) ideal, such as Jones’ definition that it is the capacity to endure as a result of the “fullest possible development of the organism” [22]. Krapf offered a less idealistic definition—as the ability to maintain a (dynamic) psychic equilibrium with ego and reason predominating [23]. In 1982, Joseph attempted to integrate previous attempts at defining normality by describing it as what is average or expectable, as defined by research or clinical experience [24]. Some of these concepts are touched on in the definition of *normal* in the *Critical Dictionary of Psychoanalysis*, though Rycroft also adds the idea that a “norm is that member of a class by comparison with which other members are described” [25].

Some of these psychoanalytic concepts of normality are more socially oriented than Freud’s definition. They have probably contributed, along with related work such as Bion’s research with groups, to an acceptance of psychoanalysis by some psychologists as “a form of social psychology” ([5], p. 111). Although Foucault’s original criticism of Freud may have been misplaced for the reasons stated earlier, the subsequent direction of psychoanalytic thought has established the potential for some renewed Foucault-style criticism. An early example of this can be seen in Thibaut’s criticism of Glover’s concept of social adaptation, saying that it leads to compartmentalisation, fails to recognise the possibility that abnormalities can occur on a mass basis, is difficult to validate, and fails to do justice to the societal ‘rebel’ who is, in fact, more normal than the abnormal mass [26]. 

### 2.2. Foucault and Analytical Psychology

The relation of analytical psychology to Foucault over time is almost a mirror image to psychoanalysis. Although Foucault’s early criticism of Freud’s concept of normality was misguided, later developments muddy the picture and raise issues about which Foucault might have had some valid points. Jung’s early work, however, is based on the type of approach that Foucault criticised, and it is Jung’s later theories that muddy the picture and move analytical psychology away from definitions of normality that are based on social norms.

Throughout most of his working life, Jung recognised the role played by collective norms [27]. He used them in his early work on word associations, first conducting experiments on normal subjects so he could subsequently distinguish pathological results [28]. This was an overt example of the process of normalisation that Foucault had suggested was covertly part of Freud’s practice. In later years, however, Jung took a more individually-oriented view of normality, rejecting Adler’s socially-oriented normalisation [29] because of the depreciating impact it has on the unconscious. He also objected to statistical averages because “individual exceptions...are murdered by statistics” [30], though he still saw a need for the notion of “average functioning” [31]. He held both views, not through inconsistency but because they were aspects of one of the “great number of antinomies...required to describe the nature of the psyche satisfactorily” [32], the universal and the individual. Although Jung began his career using the process of normalisation of which Foucault was so critical, as time went on Jung’s position became more aligned with Foucault, e.g., observing the fact of normalisation whilst recognising the damaging impact it could have on the individual.

## 3. The Concept of Normality in Analytical Psychology

### 3.1. Differences between Freud and Jung

By comparison with mainstream psychology or psychoanalysis, the concept of normality within analytical psychology remains relatively undeveloped. Although there is a body of work that is relevant to normality—such as that which describes the “normal” archetypal stages of life (e.g., [33]), applies Jung’s ideas to the social sciences (e.g., [34]), or discusses normality in opposition to pathology (e.g., [35]), there are three problems with it. Firstly, discussions that utilise analytical psychology tend not to focus on the mainstream of normal life but on the fringes. For example, the Jungian analyst Marian Woodman seems to position analytical psychology as not being relevant to “happy carrot” normal people [36], and research into Jung’s theories in mainstream academic psychology is very limited. Secondly, the existing body of work is missing a cornerstone of definition, because the post-Jungian development of the concept of normality itself is relatively limited (e.g., [37]). For example, there is no entry for “normal” in *A Critical Dictionary of Jungian Analysis* [38], and Samuels’ summary in *Jung and the Post-Jungians* cites mostly the work of psychoanalysts [39]. And, thirdly, the focus of the existing body of work is overwhelmingly on the relationship between the ego and the unconscious. In psychoanalysis, the emergence of ego psychology broadened its scope, leading to a greater awareness of the need to adapt to external reality, rather than focusing primarily on the ego’s relation to unconscious processes. There has been no parallel in analytical psychology because the vast majority of development has been based on the relationship of the ego to the Self. As a result, the relationship between the ego and the persona has been neglected. Although this, in part, reflects Jung’s emphasis on the unconscious, he nevertheless had some radical and important things to say about normality, the relationship between the ego and the persona, and the application of analytical psychology to the normal population. To begin any substantive discussion on the subject of normality, therefore, one has to start by going back and reviewing the oft-overlooked aspects of Jung’s original work.

There are some overlaps between Jung’s notions and those of psychoanalysis, such as the idea of there being an “average course of events” [31]. However, whilst Jung recognised the role of inner conflict in creating neurosis ([1], p. 211), he did not share Freud’s view that normality was freedom from such conflict. Jones’ ideal state of development, or Freud’s “ideal fiction” [40], corresponds to Jung’s state of wholeness that is the final goal of individuation. Therefore, although the word *normal* is used in both psychoanalysis and analytical psychology, there are two separate meanings being used. Freud’s “normal” equates to Jung’s “individuation”, and Jung’s concept of “normal” can include a group that is opposite to Freud’s—those who lack any significant development of consciousness [41]. Also, whilst Freud’s normality is a fiction because it is unattainable, Jung says “the normal man is a fiction” [42] because there is no individual who is identical to the collective norms, *i.e.*, that “every individual is an exception to the rule” [41].

### 3.2. Bi-Directional Adaptation

In Jung’s view, “normality is a most relative conception” ([31], p. 210), a dynamic balance between the inner and outer worlds. Achieving this balance is central to analytical psychology:

[The] main purpose [of analytical psychology] is the better adaptation of human behaviour, and adaptation in two directions (illness is faulty adaptation)...to external life—profession, family, society—and secondly to the vital demands of his own nature...to bring it to the right pitch of development.

[43]

Jung used the metaphor of specific gravity to suggest that each individual has their own natural level of adaptation between the two worlds [42]—an image that can be clarified by likening the ego to a hydrometer, which sits at a certain height between a fluid and a gas depending on the fluid’s specific gravity. Individuals can become neurotic when they are unable to find the right position between the two, e.g. not having a desired level of adaptation to the context, or being unfulfilled by it because of an inner disposition ([29], p. 70). This means that, in broad terms, there are two types of neurosis either side of normality, “collective people with underdeveloped individuality [or] individualists with atrophied collective adaptation” ([32], p. 7):

The first type is...the kind of neurosis which...is a form of maladjustment based on personal weakness...The reason for [the second type’s] neurosis seems to lie in their having...an overplus for which there is no adequate outlet.

[44]

This adaptation is not just a function of the individual psyche and immediate context, *i.e.*, adjustment to the conditions of the moment, but it also needs to take account of both historical trends and emerging possibilities in the two domains of adaptation:

In the eyes of the Extravert...adjustment...must seem like complete adaptation [but] the objective situation...can quite well be temporarily or locally abnormal...Adjustment is not adaptation: adaptation requires far more than merely going along smoothly with the conditions of the moment...It requires observance of laws more universal than the immediate conditions of time and place.

[45]

Therefore, normality in analytical psychology is a psychic ecology in which there is a sustainable balance between the inner and outer worlds. This definition does not require being free from complexes, and there are many psychic processes that Jung viewed as normal that Freud and others viewed as pathological. For example, it is normal for the psyche to split and for individuals to fall painfully or permanently under the control of complexes:

Complexes are the normal foci of psychic happenings, and the fact that they are painful is no proof of pathological disturbance. Suffering is not an illness, it is the normal counter pole to happiness.

[46]

The presence of autonomous complexes is not itself pathological, since normal people, too, fall temporarily or permanently under their domination. This fact is simply one of the normal peculiarities of the psyche.

[47]

The sophistication of Jung’s understanding of normality is illustrated by his oxymoronic phrase “normal peculiarities”. Normality is not a particular psychic state but an overall pattern that embraces a wide range of emerging psychic states, including peculiar ones. This is similar to observations in statistics, where a normal distribution is not just a single, average result but a pattern that emerges from a wide range of values—including ones at the extreme of the curve. A practical example of this is Christopher Hauke’s argument that fragmentation and narcissism can be seen in a positive light, as motors that drive the process of individuation [48]. For some people fragmentation can be viewed as normal, as it is expectable in the overall context of a psyche working towards its need of individuation through therapy. For others, fragmentation is not expectable in daily life and it might disturb their psychic balance by interfering with the basic level of collective adaptation that is required for individuation to progress ([41], p. 449).

### 3.3. Conscious Conflicts and Projections

Jung’s definition of normality has a number of significant and oft-overlooked implications. In Jung’s scheme, one of the key distinctions between neurotics and normal people is the nature of the conflicts they experience. When someone is neurotic, the ego has not found the individual’s natural level between the two domains of adaptation so, in terms of the metaphor of specific gravity, the (unconscious) fluid tries to restore balance either by pushing the ego up to better adapt to the context, or by pulling it back down into the unconscious. This means that, for a neurotic, there is some form of conflict between the ego and the unconscious, which might only be detectable through unexplained symptoms such as chronic anxiety. For normal people, however, that positional conflict between consciousness and the unconscious is not there. They still experience conflicts, as these are a part of normal life, but to the individual they seem to have a different nature:

The mental and moral conflicts of normal people [are of] a somewhat different kind: the conflicting opposites are both conscious.

[49]

For normal people, there are still differences between consciousness and the unconscious, and there are still conflicting aspects within both the conscious and unconscious psyche. However, they experience them in different ways to neurotics, by being unaware of those conflicts in oneself, and/or experiencing them as a conscious conflict through projection. This may be a reason why theories such as Isabel Briggs Myers’ version of psychological type are so popular—the normal person can experience and deal with *otherness* unconsciously by seeing oneself as one personality type and another person as a different type. The theory enables them to project their own unconscious intra-psychic conflict into what are viewed consciously as inter-psychic differences between people.

Another illustration of Jung’s “normal” condition, and the unconscious compensation that goes with it, can be seen in Alexander Solzhenitsyn’s satirical reflection on where good and evil are placed in relation to oneself and others:

If only there were evil people somewhere insidiously committing evil deeds, and it were necessary only to separate them from the rest of us and destroy them. But the line dividing good and evil cuts through the heart of every human being. And who is willing to destroy a piece of his own heart?

[50]

This illustrates how normal people deal with the presence of both good and evil within one’s own psyche. If it is untenable to recognise the presence of evil within oneself, or to deal with the internal conflict that this creates, it is dealt with unconsciously. The individual takes a one-sided approach, seeing the good in oneself whilst excluding from consciousness the unacceptable aspects. These evil parts of one’s own personality then “sink into the unconscious, where they form a counter-weight to the conscious orientation” ([41], p. 419). This unconscious compensation is akin to the balancing effect of the keel of a boat that is leaning to one side in the wind. It can sometimes take a benign form, for example in dreams that can play a compensatory role for normal people even when the dreamer does not understand its meaning [51]. Also, normal people may be shielded from the potential negative effects of unconscious conflict by myths and symbols [52] which can be effective even when they are not understood [53]. 

However, compensation often takes the form of projection, which can sometimes have damaging side-effects. For example, if we take a one-sided, conscious view of ourselves as wholly good, then the psyche compensates by finding “willing carriers of our projections” [53], projecting the evil into them, and then sustaining a well-distributed web of reciprocal projections in which relationships are largely imaginary, * i.e.*, more projection than objective reality. To maintain the split between good and evil, these negative projections have to “settle outside our circle of intimate relationships” [54], a principle that applies at all levels, even to international relations. An example of how this works in practice can be seen in the prediction made in 1989 by the Jungian analyst Jerome Bernstein, who used this principle of compensatory projection to predict the rise of terrorism from the Middle East. His argument was that the changing relationship between the US and USSR would mean that the latter could no longer carry the collective projections of the former. Another group of nations would therefore have to emerge as carriers of the collective projections of the US. This displacement of projections was epitomised 15 years later in George W. Bush’s labelling of Iran, Iraq and North Korea as the “axis of evil”.

Projections are not inherently a bad thing, because they can be vital to maintaining psychic equilibrium in the person or society who is projecting, and raising awareness of those projections can become “an impediment to our relations with others” [54]. Normal people come through times of difficulty unconsciously ([1], p. 129), but this can lead to problems when negative projections are placed on people over whom one has some power or influence, or with whom one needs to have a good working relationship. When these projections aggregate at a cultural level, and can be acted out using 20^th^ century technology, they pose a significant risk to whole groups or societies:

The only real danger that exists is man himself. He is the great danger, and we are pitifully unaware of it. We know nothing of man, far too little. His psyche should be studied, for we are the origin of all coming evil.

[55]

Man’s worst sin is unconsciousness…and in all seriousness [we need to] seek ways and means to exorcize him, to rescue him from possession and unconsciousness, and make this the most vital task of civilisation.

[56]

For Jung, unconsciousness and the excessive one-sidedness that can accompany it were amongst the greatest threats to civilisation. The increase of consciousness by integrating unconscious contents (*i.e.*, individuation) was therefore a moral imperative that affects not only individuals but also international and cultural relations.

### 3.4. Individual-Collectivity Spectrum

One of the common misperceptions about analytical psychology is that individuation is its central goal, e.g., the Wikipedia page on Jungian or analytical psychology explicitly states: “The overarching goal of Jungian psychology is the attainment of self through individuation” [57]. At the time of writing this paper, that page goes on to describe neurosis in terms of adaptation to the Self (*i.e.*, the central archetype in the inner world) and the goal of psychotherapy as re-establishing a healthy relationship with the unconscious. However, although individuation is very significant aspect of Jung’s theory, it is not the only goal for normal people—it is not even the only goal in therapy ([41], p. 449). Neurosis can also arise from the other direction of adaptation, *i.e.* the context or environment. Jung gave some specific examples of this, such as an American businessman who became neurotic because he could not adapt to retirement after running a business for his entire life ([1], pp. 50–52), or how a young person can sometimes be cured of a neurosis through a new start, if it is rich in possibilities. These examples are in line with contemporary research, such as that showing the importance of continuity of social roles in retirement [58].

Promoting individuation as the sole aim of analytical psychology creates some problems in applying Jung’s theory to normal (non-neurotic) people. Jung’s primary goal, as stated earlier, is two-way adaptation, to both the inner and outer worlds, and a main theme that runs through all aspects of his theory is dealing with the problem of opposites through the transcendent function [59]. An exclusive focus on individuation presents his theory, ironically, in a one-sided way. It misses the point that individuation and collectivity are themselves a pair of opposites, and it neglects applications of analytical psychology at one of the poles (collectivity) that are relevant to many in the normal population:

There are countless people who are not only collective [but whose] ambition [is] to be nothing but collective.

([32], p. 7)

Individuation and collectivity are a pair of opposites, two divergent destinies...The individual is obliged by the collective demands to pursue his individuation...Anyone who cannot do this must submit directly to the collective demands, to the demands of society.

[60]

The choice between individuation and collectivity is not a binary one, because both are involved at both ends of the individuation-collectivity spectrum. Working towards individuation “presupposes and includes collective relationships” [39], p. 77), and pursuing the goal of collectivity also includes an element of individuation. In defining the main goal for analytical psychology, Jung did not suggest that everyone should work towards individuation, but rather that they should “get involved in the very fate for which they were suited” ([1], p. 149). Furthermore, he gave strong warnings against forcing someone into anything other than their own destiny. For example, it can be dangerous to artificially bring unconscious contents to the surface ([27], p. 153), akin to “digging an artesian well and running the risk of stumbling on a volcano” ([1], p. 114), and it could lead to such a “disastrously wrong turning” [61] that he would be the first to hold back. He also gave a warning in the other direction, against the collectivisation of those whose destiny is individuation, because “the suppression of individuality through the predominance of collective ideals and organisations is a moral defeat for society” [62].

Jung’s theory already contains many inherent problems, paradoxes and contradictions—such as the dichotomy that it can be in the individual’s best interests to remain unconscious but in society’s interest to become more conscious. However, if the goal of Jung’s theory is presented only as a psychology of individuation and of rapprochement with the unconscious, then this creates an additional problem. It positions analytical psychology as not particularly relevant to normal people, it overlooks societal/contextual causes of neurosis, and it means we may miss some of the potential insights and applications that it has to offer mainstream psychology and everyday living. It also means we are less likely to resolve the dichotomy discussed above—of the individual *vs.* societal benefits of consciousness remaining the same or increasing.

### 3.5. Personal and Collective Awareness

One of the perhaps surprising implications of Jung’s definition of normality is the relationship between the individuation-collectivity spectrum and an increase in consciousness. Raising awareness of the personal unconscious makes one more collective, whilst raising awareness of the collective unconscious makes one more individual. Also, the first type of awareness tends to lead naturally to the latter, because the two types of content are “inextricably merged” ([39], p. 139):

[R]aising the personal unconscious to consciousness...[makes one] less individually unique, and more collective.

([1], p. 148)

The ego-consciousness is at first identical with the persona...[T]hrough the analysis of the personal unconscious, the conscious mind becomes suffused with collective material which brings with it the elements of individuality.

([1], p. 158)

Jung’s first statement, about becoming more collective, may seem surprising to some, but Jacobi makes a similar point when she describes “analysis of the personal unconscious” as “adjustment to external reality” [63]. Although this may seem counter-intuitive, it can perhaps be explained by considering what happens in the process of 360-degree feedback, which is now a normal occurrence in many business and organisational contexts. In this process, colleagues at all levels—bosses, peers, and subordinates—give an individual feedback on their behaviour. The common themes from this feedback are very likely to be dominated by the collective cultural values of the organisation, and how well the individual’s behaviour fits with those values. As a result, people receiving such feedback are likely to change their attitudes and behaviour to be more in line with the cultural norms, *i.e.*, become more collective. However, some feedback may also contain comment on the person’s unique potential and deeper aspects of his/her individual personality. If so, this may lead towards a process of individuation, which may result in the person making a more unique contribution to the development of the organisation.

The nature of this collectivisation process, based on awareness of the personal unconscious, has not been investigated in any depth within analytical psychology. The most relevant aspects of Jung’s writings are those that discuss the persona, which is a portion of the collective psyche. Collectivisation doesn’t necessarily involve analysis of the persona, for “when we analyse the persona we strip off the mask” ([1], p. 158). Rather it involves the formation of a “properly developed persona” ([61], p. 199) for, as Casement notes, “the development of a well-functioning persona is an essential task for any individual” [64] and it can provide protection for the ego and psyche. The development of a healthy persona will eventually lead back towards individuation because it involves a differentiation of ego and persona. That is, in the public life of the persona one becomes more collective, but in the private life of the ego, which may be apparent only in contained relationships such as marriage, one starts to individuate.

From the perspective of individuation, “fundamentally the persona is nothing real: it is a compromise between individual and society as to what a man should appear to be” ([1], p. 158) and it can present an obstacle to growth (e.g., see [65]). However, for the normal population, Jung’s work on the persona sits at an important point of intersection between analytical psychology and social psychology, which views the persona as vital and providing a channel through which the normal person can find meaning [66], or through which meaning emerges [67].

## 4. Conclusions

It is likely that people who seek Jungian analysis, or otherwise have a personal interest in analytical psychology, tend to be interested in their own individuation. However, if analytical psychology is to be applied to the normal population then the goal is a little different—it is to help them find their own natural balance on the spectrum between individuation and collectivity, and then to increase consciousness at their own natural pace if it suits their individual destiny. For collective people there may only be one direction of development—they are defined by their social roles, and they continue to play that role (or similar ones) throughout the whole of life. For others, whose natural destiny is individuation, there may be two phases. The first is a move towards collectivity through increased awareness of the personal unconscious. This then leads naturally back towards the individuation end of the spectrum, through the emergence of contents from the collective unconscious. These two phases are related to the two movements of individuation described by Stein [68] and they highlight some of the key problems in applying analytical psychology to the normal population. For example, it is a truism to say that, for society to function, there has to be a degree of collectivity, but too much emphasis on collectivity can suppress the individual. Another problem is the dual impact of unconsciousness, which can protect the individual from intra-psychic conflicts, but can also damage other people and society through projection. Associated with unconsciousness is one—sidedness-a key feature of the first movement of individuation—but too much one-sidedness can become an obstacle to progress, preventing the reconciliation of opposites and the integration of the unconscious.

The key challenge in applying analytical psychology to normal people, therefore, is to find the right balance between individuation and collectivity in a way that both serves society and meets each individual’s needs and destiny. This requires a culture that values all parts of the collectivity-individuation spectrum, encourages individuals to find their natural place on it, and enables an ongoing progression whilst avoiding the problems of excessive one-sidedness, unconsciousness, and the analytic risks to the individual. Whereas neurotics are forced by their neurosis to become more conscious ([1], p. 272), an increase in consciousness in normal people can only be pursued through a *natural* process of transformation within the individual [69]. Meeting this challenge requires more research and development, particularly in the area of the persona. Whereas analytical psychologists tend to emphasise the *ego-self axis*, for the normal population the *ego-persona axis* is also very significant because of their direct involvement in collectivity. Yet neither axis represents a complete picture, so further development of the concept of normality in analytical psychology needs to be based on a healthy triangular relationship that affords value to all three.

## References

[1] Jung C.G. (1966). On the Psychology of the Unconscious. Two Essays on Analytical Psychology, Collected Works 7.

[2] Jung C.G. (1977). Foreword to Von Koenig-Fachsenfeld: “Wandlungen des traumproblems von der romantic bis zur gegenwart”. The Symbolic Life, Collected Works 18.

[3] Shamdasani S. (2003). Jung and the Making of Modern Psychology.

[4] Jung C.G. (1977). Address on the Occasion of the Founding of the C.G. Jung Institute, Zurich, 24 April, 1948. The Symbolic Life, Collected Works 18.

[5] Wetherall M. (1996). Identities, Groups and Social Issues.

[6] Sherif M. The Psychology of Social Norms.

[7] Lewin K. Group Decision and Social Change.

[8] Foucault M. (1991). Discipline and Punish: The Birth of the Prison.

[9] Foucault M., Rabinow P. (1984). Madness and Civilization. The Foucault Reader.

[10] Craig E. (2005). The Shorter Routledge Encyclopaedia of Philosophy.

[11] Whitebook J. (1999). Freud, Foucault and ‘the Dialogue with Unreason’. J. Philos. Soc. Critic..

[12] Gutting G. (2005). The Cambridge Companion to Foucault.

[13] Freud S., Crick J. (1999). The Interpretation of Dreams.

[14] Freud S. (1901). The Psychopathology of Everyday Life: Forgetting, Slips of the Tongue, Bungled Actions, Superstitions and Errors. The Standard Edition of the Complete Psychological Works of Sigmund Freud.

[15] Freud S. (1926). Psychoanalysis: Freudian School. Encyclopaedia Britannica.

[16] Freud S. (1895). Letter from Freud to Fliess, May 25, 1895. The Complete Letters of Sigmund Freud to Wilhelm Fliess.

[17] Freud S. (1890). Psychical (or Mental) Treatmen. The Standard Edition of the Complete Psychological Works of Sigmund Freud, Volume VII.

[18] Kubie L.S. (1954). The Fundamental Nature of the Distinction between Normality and Neurosis. The Psychoanalytic Quarterly, Volume 23:167–204.

[19] Offer D., Sabshin M. (1973). Normality.

[20] Glover E. (1932). Medico-Psychological Aspects of Normality. Br. J. Psychol..

[21] Gitelson M. (1954). Therapeutic Problems the Analysis of the ‘Normal’ Candidate. Int. J. Psychoanal..

[22] Jones E. (1942). The Concept of a Normal Mind. Int. J. Psychoanal..

[23] Krapf E.E. (1961). The Concepts of Normality and Mental Health in Psycho-Analysis. Int. J. Psychoanal..

[24] Joseph E.D. (1982). Presidential Address-Normal in Psychoanalysis. Int. J. Psychoanal..

[25] Rycroft C. (1995). Critical Dictionary of Psychoanalysis.

[26] Thibaut J.W. (1943). The Concept of Normality in Clinical Psychology. Psychological Review.

[27] Jung C.G. (1981). The Significance of the Unconscious in Education. The Development of Personality, Collected Works 17.

[28] Jung C.G. (1973). The Associations of Normal Subjects. Experimental Researches, Collected Works 2.

[29] Jung C.G. (1966). Problems of Modern Psychotherapy. The Practice of Psychotherapy, Collected Works 16.

[30] Jung C.G. (1970). Flying Saucers: A Modern Myth. Civilization in Transition, Collected Works 10.

[31] Jung C.G., Adler G. (1973). Letter to Smith Ely Jellife. C.G. Jung Letters Volume I 1906–1950.

[32] Jung C.G. (1966). Principles of Practical Psychotherapy. The Practice of Psychotherapy, Collected Works 16.

[33] Neumann E. (1954). The Origins and History of Consciousness.

[34] Progoff I. (1959). Depth Psychology and Modern Man.

[35] Noll R. (1989). Multiple Personality, Dissociation and C.G. Jung’s Complex Theory. J. Anal. Psychol..

[36] Woodman M., Mellick J. (2001). Coming Home to Myself: Reflections for Nurturing a Woman’s Body and Soul.

[37] Mizen R. (1995). The Primitive and the Pathological (Review). J. Anal. Psychol..

[38] Samuels A., Shorter B., Plaut F. (1986). A Critical Dictionary of Jungian Analysis.

[39] Samuels A. (1986). Jung and the Post Jungians.

[40] Freud S. (1937). Analysis Terminable and Interminable. The Standard Edition of the Complete Psychological Works of Sigmund Freud, Volume XXIII.

[41] Jung C.G. (1991). Psychological Types, Collected Works 6.

[42] Jung C.G. (1981). Marriage as a Psychological Relationship. The Development of Personality, Collected Works 17.

[43] Jung C.G. (1981). Analytical Psychology and Education. The Development of Personality, Collected Works 17.

[44] Jung C.G. (1966). Fundamental Questions of Psychotherapy. The Practice of Psychotherapy, Collected Works 16.

[45] Jung C.G. (1991). Psychological Types, Collected Works 6.

[46] Jung C.G. (1966). Psychotherapy and a Philosophy of Life. The Practice of Psychotherapy, Collected Works 16.

[47] Jung C.G. (2003). On the Relation of Analytical Psychology to Poetry. The Spirit in Man, Art and Literature, Collected Works 15.

[48] Hauke C. (1995). Fragmentation and Narcissism. J. Anal. Psychol..

[49] Jung C.G. (1977). Techniques of Attitude Change Conducive to World Peace. The Symbolic Life, Collected Works 18.

[50] Solzhenitsyn A.I. (1985). The Gulag Archipelago, 1918–1956: An Experiment in Literary Investigation.

[51] Jung C.G. (1969). On the Nature of Dreams. The Structure and Dynamics of the Psyche, Collected Works 8.

[52] Jung C.G. (1967). Symbols of Transformation, Collected Works 5.

[53] Jung C.G. (1968). On the Psychology of the Trickster Figure. The Archetypes and the Collective Unconscious, Collected Works 9i.

[54] Jung C.G. (1969). General Aspects of Dream Psychology. The Structure and Dynamics of the Psyche, Collected Works 8.

[55] Jung C.G. (1977). The “Face to Face” Interview. C.G. Jung Speaking.

[56] Jung C.G. (1968). The Phenomenology of the Spirit in Fairytales. The Archetypes and the Collective Unconscious, Collected Works 9i.

[57] Analytical Psychology. http://en.wikipedia.org/wiki/Analytical_psychology.

[58] Stokes G. (1992). On Being Old.

[59] Miller J.C. (2004). The Transcendent Function.

[60] Jung C.G. (1977). Adaptation, Individuation, Collectivity. The Symbolic Life, Collected Works 18.

[61] Jung C.G. (1966). The Relations between the Ego and the Unconscious. Two Essays on Analytical Psychology, Collected Works 7.

[62] Jung C.G. (1966). The Structure of the Unconscious. Two Essays on Analytical Psychology, Collected Works 7.

[63] Jacobi J. (1958). The Process of Individuation. J. Anal. Psychol..

[64] Casement A. (2010). Persona. Encyclopaedia of Psychology and Religion.

[65] Hudson W.C. (1978). Persona and Defence Mechanisms. J. Anal. Psychol..

[66] Perlman H.H. (1986). Persona: Social Role and Personality.

[67] Miell M., Dallos R. (1996). Social Interaction and Personal Relationships.

[68] Stein M. (2006). Principle of Individuation: Toward the Development of Human Consciousness (Polarities of the Psyche).

[69] Jung C.G. (1968). A Study in the Process of Individuation. The Archetypes and the Collective Unconscious, Collected Works 9i.

